# Selective embolization for hypervascular metastasis from differentiated thyroid cancer: a case series

**DOI:** 10.1186/1752-1947-8-405

**Published:** 2014-12-04

**Authors:** Hee Young Son, Soo-Youn An, Eui Young Kim, Sang Bu Ahn, Byung Chul Lee

**Affiliations:** 3 Department of Otorhinolaryngology, Thyroid/Head & Neck Cancer Center, Dongnam Institution of Radiological & Medical Sciences, 40 Jwadong-gil, Jangan-eup, Gijang-gun, Busan, South Korea; 4 Department of Endocrinology, Thyroid/Head & Neck Cancer Center, Dongnam Institution of Radiological & Medical Sciences, 40 Jwadong-gil, Jangan-eup, Gijang-gun, Busan, South Korea; 5 Department of Radiology, Thyroid/Head & Neck Cancer Center, Dongnam Institution of Radiological & Medical Sciences, 40 Jwadong-gil, Jangan-eup, Gijang-gun, Busan, South Korea; 6 Department of Otorhinolaryngology, Thyroid/Head & Neck Cancer Center, Korea Cancer Center, 75 Nowon-ro, Nowon-gu, Seoul, South Korea

**Keywords:** Embolization, Neoplasm metastasis, Thyroid cancer

## Abstract

**Introduction:**

The technique of selective embolization has been applied for years in the treatment of vascular anomalies, severe hemorrhage, and for benign and malignant tumors. Some hypervascular skeletal metastases are prone to massive hemorrhage.

**Case presentation:**

We describe the cases of two patients with thyroid carcinoma presenting with neuromuscular symptoms due to large skeletal metastases in the shoulder and sternum respectively. Pre-operative percutaneous selective catheterizations of the arteries feeding the metastatic tumors were performed, followed by infusion of gelfoam. The procedures were technically successful in both patients without adverse effects or bleeding. Complete resections of the skeletal metastases were then performed without substantial bleeding.

**Conclusion:**

Selective embolization is an effective treatment for bony metastases from thyroid cancer.

## Introduction

Well-differentiated thyroid carcinoma with bony metastases to the sternum or clavicle is uncommon. Patients with metastatic disease from thyroid carcinoma have a poor prognosis in general. However, adjuvant therapy may reduce the tumor burden in some patients, offering a survival or palliative benefit. Treatment modalities for patients with metastatic thyroid cancer include surgical excision, radioactive iodine (RAI) therapy with I-131, external beam radiotherapy, or novel therapies through recruitment into clinical trials. Large primary tumors and bony metastases are predictive of a poor response to RAI therapy. Complete surgical resection of isolated symptomatic metastases has been associated with improved survival and should be considered. For skeletal metastases in particular, surgery is recommended for symptomatic as well as asymptomatic lesions in weight-bearing extremities [1, 2].

Numerous studies have indicated that a large proportion of skeletal metastases from thyroid carcinoma are hypervascular, with bleeding consequently the most common complication encountered during surgery of these lesions. A few studies recommend embolization before resection of these bony metastases [2–4].

Bone metastases from thyroid carcinoma to the sternum and clavicle are uncommon and have been reported only rarely. We present two cases of thyroid cancer with bony metastases to the sternum and clavicle, and describe management with pre-operative embolization followed by definitive surgery.

## Case presentation

### Case 1

**Figure 1:**
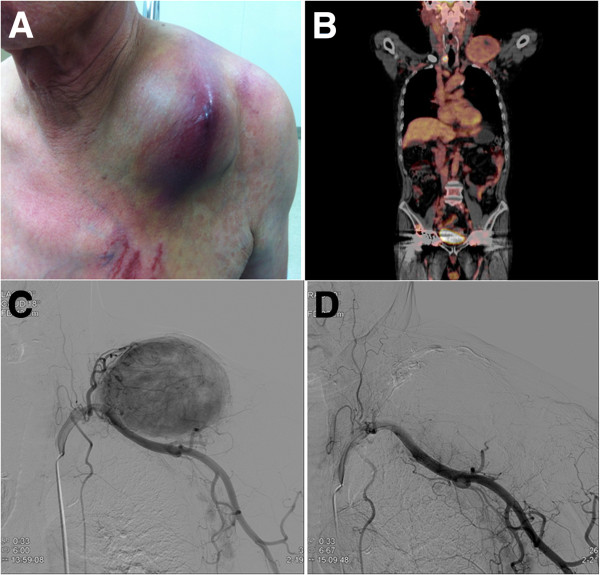
**A 71-year-old man presented with a painful enlarging mass over the left clavicle. (A)** Physical examination. **(B)** Positron emission tomography reveals a right thyroid tumor with a left clavicular metastasis. **(C)** Angiographic image from selective catheterization of the left subclavian artery. **(D)** Follow-up angiography after embolization shows no residual tumoral blush.

A 71-year-old man presented to our center with a three-year history of a painful, progressively enlarging mass over his left clavicle and shoulder (Figure 1A). Physical examination revealed a 10×10cm mass of his left clavicle extending to involve his left shoulder. A positron emission tomography-computed tomography showed a right thyroid tumor with bone metastases to his left clavicle and shoulder (Figure 1B). There were also multiple indeterminate nodules in the isthmus and left lobe of his thyroid gland on sonography (Figure 1C). A diagnosis of metastatic thyroid carcinoma was made after an incisional biopsy of the left clavicular mass (Figure 1D). There was heavy bleeding during the biopsy, with a large amount of post-procedural hemorrhagic drainage.

On the day prior to surgery, selective percutaneous catheterization of the arteries feeding the metastatic lesion was performed, followed by infusion of absorbable gelatin sponge particles (Cutanplast®; Mascia Brunelli Spa, Milano, Italy). The clavicular mass was fed by branches of the left subclavian artery. Additional selective micro-catheterization and embolization were performed on four feeding arteries (likely arising from the transverse cervical, suprascapular, clavicular and acromial branches of the thoracoacromial arteries) with gelfoam. There was no acute complication encountered during or following embolization.

A total thyroidectomy with wide composite resection of the large clavicular mass was performed the following day. The metastatic lesion was easily separated from his left clavicle following complete tumor devascularization and necrosis by the pre-operative embolization. The estimated blood loss was low. Our patient received post-operative RAI (I-131) therapy. His post-operative recovery was uneventful, and he has been well and pain-free since surgery.

### Case 2

**Figure 2:**
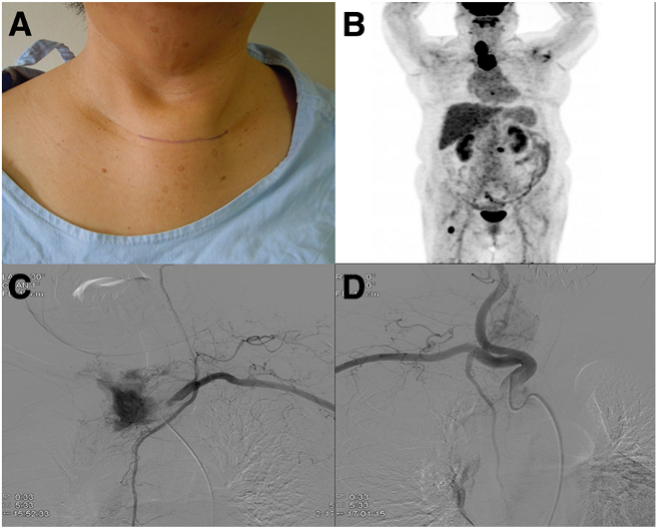
**A 64-year-old woman presented with an enlarging mass of the upper sternum. (A)** Physical examination. **(B)** Positron emission tomography showed a right thyroid tumor with a sternal metastatic lesion. **(C)** Angiographic image from selective catheterization of the left internal mammary artery. **(D)** Follow-up angiography after embolization reveals no residual tumoral blush.

A 64-year-old woman presented to our center with a painful hard mass over the upper part of her sternum. She had a history of a thyroid tumor 30 years ago but did not undergo surgery. The sternal mass was progressively enlarging and had been present for a month. On physical examination, a 4.0×3.7cm mass was found in her upper sternum extending to the region of the clavicular heads.Positron emission tomography-computed tomography revealed a 3.3cm tumor in her right thyroid gland and another 4.0cm upper sternal osteolytic mass (Figure 2B). A diagnosis of metastatic follicular carcinoma was made via fine-needle aspiration cytology of the sternal mass.

Catheter angiography was performed prior to embolization to identify the feeding vessels supplying the metastatic lesion and to determine the feasibility and safety of embolization. These arteries were then catheterized, the choice of catheter depended upon the size of the feeding vessels. Infusion of absorbable gelatin sponge particles (Cutanplast®; Mascia Brunelli Spa) was performed following selective catheterization of the feeding vessels. The sternal metastatic mass was fed by branches of the internal mammary arteries bilaterally. There was no immediate complication encountered during or after embolization.

Our patient subsequently underwent a total thyroidectomy, with partial sternectomy for the metastatic lesion using a sternal saw. Sternal reconstruction was performed with bone cement and Malex mesh. The procedure was technically straightforward after the complete devascularization and necrosis of the sternal metastasis, which resulted in tumor ischemia and hypovascularity.

Our patient received post-operative RAI (I-131) therapy. No evidence of cancer recurrence or distant metastasis was detected during subsequent follow-up visits.

## Discussion

Distant metastasis occurs in 10% to 20% of well-differentiated thyroid carcinoma, most frequently to the lung and bone. RAI therapy is the gold-standard for treatment and remains a primary treatment modality for metastatic thyroid carcinoma, but poor tumor uptake of the agent can limit its usefulness. Results of ablation therapy are good with lung metastases, but bone metastases are not cured with ablation therapy alone. As such, osseous metastasis in differentiated thyroid carcinoma confers a significantly worse prognosis. Treatment options in patients with bone metastases from differentiated thyroid carcinoma are limited and mostly palliative. Other treatment modalities include surgery or external irradiation. Our report describes two cases of differentiated thyroid carcinoma with clavicular and sternal metastases that were managed successfully by surgery with pre-operative embolization. This demonstrated the effectiveness of embolization in the management of isolated skeletal metastasis from differentiated thyroid carcinoma [3, 5, 6].

Surgery is indicated as first-line therapy in individual patients and should be advocated in cases of solitary bone metastasis. This therapeutic approach occasionally remains the only chance of offering a long-term cure with an improved quality of life and prolonged survival in cases where isolated metastases are amenable to resection. Furthermore, with multiple metastases or local recurrent disease, resection of skeletal metastases can allow for more efficient radio-iodine treatment, making surgical removal of resectable skeletal metastases a valuable complement to RAI therapy. Zettinig *et al*. identified surgical extirpation of bone metastases as a significant prognostic factor associated with improved survival in patients with distant metastases limited to the bones. This study concluded that in patients without additional extraskeletal distant metastases, radical surgical extirpation of bone metastases may be associated with improved survival [1–3, 6, 7].

Bleeding is the most common complication encountered at operation for these skeletal metastases. More than 60% of skeletal metastases, including those to the spine, demonstrate hypervascularity and neovascularity. Efforts have been made to decrease the incidence of intra-operative hemorrhage [8, 9].

Embolization was first reported by Dr Frieda Feldman in 1975 as a useful adjunct in the management of selective bone tumors. Many studies have since reported on selective and super-selective intra-arterial embolization as an effective treatment with a rapid reduction in pain and tumor volume of primary bone and soft tissue tumors, including bony metastases from various primary malignancies. Embolization provides for devascularization, size reduction, calcification of margins, and pain relief; it can be palliative or adjunctive, as well as primary or serial [2, 6–9].

The main purpose of embolization is to occlude as much of the tumor vascular supply as possible, yet avoiding damage to adjacent normal tissue. The outcomes range from complete tumor devascularization and necrosis to various degrees of ischemia and hypovascularity. This would ideally lead to tumor shrinkage, reduced intra-operative bleeding, and clearer delineation of margins between the tumor and surrounding tissues, thus allowing for easier resection. Decreased intra-operative bleeding is particularly important in patients with rare blood groups or in those prone to transfusion reactions. This is crucial considering that high transfusion requirements in tumor surgery are frequently complicated by depletion of clotting factors and coagulopathy that may further exacerbate intra-operative bleeding, while blood salvage techniques are contraindicated because of the risk of dissemination of tumor cells. In addition, in large and unresectable tumors, decreasing the volume of viable tumour tissue, destroying the tumor tissue, or at least inhibiting tumor growth will decrease the required doses of radiotherapy and chemotherapy [2, 6, 9].

Therapeutic embolization of osseous metastases has been previously reported in a small number of patients with a variety of tumors. This procedure has also been shown to result in a rapid resolution of associated neurological symptoms. In our patients, it was believed that selective arterial embolization might be inadequate in controlling intra-operative bleeding, not only due to the size of the metastases but also because of resistance to pharmaceutical antiangiogenic manipulations. Thus, the treatment plan of these patients included surgery closely following embolization [2, 6, 9, 10].

Gelfoam or gelatin sponge is a dissolvable sponge-like material that has been used for many years in surgery. It comes in small, flat, rectangular blocks that can be either cut with scissors into elongated rectangles or rolled into pledgets and injected via diagnostic catheters or microcatheters. Alternatively, the material can be cut into small cubes and mixed vigorously in a syringe to form a slurry (two syringes connected via a stopcock works well). Gelfoam is considered a temporary occluding agent, with the occluded vessel re-canalizing in two to four weeks, although the evidence for the exact timeframe is limited [7, 9, 11].

## Conclusion

In view of the insensitivity of bone metastasis to RAI therapy, the authors believe that pre-operative embolization followed by definitive surgery of solitary osseous metastasis from thyroid carcinoma is a safe and effective treatment option, even in technically demanding anatomical locations.

## Consent

Written informed consent was obtained from the patients for publication of this case report and any accompanying images. A copy of the written consent is available for review by the Editor-in-Chief of this journal.
